# Epidemiology of Endocrine Dysfunctions in Pediatric Patients with Previous Central Nervous System Infection: A Scoping Review of the Literature

**DOI:** 10.3390/children11070794

**Published:** 2024-06-28

**Authors:** Giorgio Sodero, Clelia Cipolla, Laura Martino, Carolina Gentili, Claudia Rendeli, Danilo Buonsenso

**Affiliations:** 1 Institute of Pediatrics, Università Cattolica del Sacro Cuore, 00168 Rome, Italy; giorgio.sodero01@icatt.it (G.S.); martino.laura96@gmail.com (L.M.); carolina.gentili01@gmail.com (C.G.); 2 Department of Woman and Child Health and Public Health, Fondazione Policlinico Universitario Agostino Gemelli—IRCCS, 00168 Rome, Italy; clelia.cipolla@policlinicogemelli.it; 3 Spina Bifida Center, Fondazione Policlinico Universitario Agostino Gemelli—IRCCS, 00168 Rome, Italy; claudia.rendeli@policlinicogemelli.it; 4 Università Cattolica del Sacro Cuore, 00168 Rome, Italy

**Keywords:** central nervous system infections, pediatric endocrinology, pediatric infectious diseases, meningitis

## Abstract

Purpose The incidence of endocrine sequelae following central nervous system (CNS) infections in pediatric age is not known. We conducted this scoping review to assess the incidence of endocrinological alterations in patients with prior CNS infections in pediatric age. Methods Our screening process included both randomized and non-randomized controlled trials. All types of observational studies, prospective and retrospective, have been included. Results Ten studies were included in our review. The cumulative number of patients in all of the studies was 211, the mean age of the population study was 4.9 (±5 years). The included papers described the following acute CNS infections: meningitis (nine studies reported eighty-five cases) and encephalitis (three studies described sixty-five cases). Two case reports and one retrospective study reported hypopituitarism as a consequence of *Mycobacterium tuberculosis* CNS infection. In five studies the patients developed endocrine comorbidities at the time of infection. Another study analyzed 49 young adults who previously had tuberculous meningitis at a mean age of 5.9 ± 5.0 years: seven patients had growth hormone deficiency, four of whom also had gonadotropin deficiency; the other three had gonadotropin deficiency, corticotropin deficiency, and mild hyperprolactinemia. Conclusion Standardized multidisciplinary follow-up and research of patients with prior CNS infection is crucial. Although pituitary reserve screening is not commonly performed in these patients, clinical and research centers should set up an endocrinological evaluation with monitoring of auxological parameters to detect the signs and symptoms of hypopituitarism early and to initiate the appropriate care in children with previous CNS infections.

## 1. Introduction

Central nervous system (CNS) infections represent a heterogeneous group of infectious diseases caused by viruses, bacteria, mycobacteria, or other pathogens with a higher incidence in neonates and in the pediatric population [1], often causing significant morbidity and presenting a high mortality rate.

According to data reported by the GBD 2019 Meningitis and Antimicrobial Resistance Collaborators [2], in 2019 there were 2.51 million new cases of meningitis worldwide, of which 1.28 million occurred in children under 5 years old. The global incidence was 192.4 per 100,000 children and 854.8 per 100,000 newborns, with significant variability based on the geographical area of origin. Bacterial infections are generally classified as meningitis, while viral infections result in neonatal encephalitis and meningoencephalitis [3]; in certain cases, bacterial meningitis could be a complication of extracranial infections such as otitis or rhinosinusitis, and can be localized, leading to brain parenchymal abscesses, or manifest as purulent empyemas, often requiring surgical drainage [4].

Regarding etiology, the bacterial forms are generally caused by *Streptococcus pneumoniae* and *Neisseria meningitidis*, with cases of *Haemophilus influenzae* decreasing due to increased vaccine coverage but remaining high in certain geographical areas [5].

Bacterial CNS infections present a high mortality rate (reported in some studies as high as 10,1% of cases [6]), thus proper management of children, with early identification of infection and appropriate pharmacological treatment, represents the primary goal.

In many children, despite hospitalization and appropriate therapies, the outcomes of bacterial infections may manifest, with the onset of neurological alterations (seizures, motor deficits, sensory deficits, and hearing or vision loss) that are typically correlated with post-inflammatory changes in brain parenchyma [7]. Among encephalitis cases, infections from Herpes Simplex Virus are the most severe as they exhibit a high prevalence (50.7%) of neurological alterations following the acute episode [8].

Less recognized are the potential endocrinological sequelae, which are generally associated with anatomical alterations of the hypothalamic–pituitary axis. Claudia Giavoli et al. [9] analyzed 19 pediatric patients with prior meningitis, highlighting that post-infectious hypopituitarism is a rare condition in children with a previous case of bacterial meningitis, likely due to a greater resilience of the pituitary gland to inflammatory stresses. Despite their findings, it is known that hypopituitarism is a potential post-infectious complication of CNS infections [10], sometimes even representing the debut symptom of the pathology in cases of severe infections such as those caused by mycobacteria [11]. Encephalitides, although they can cause severe neurosensory alterations following the episode in half of the cases, are not a common cause of hypopituitarism [8], while brain abscesses can cause hypothalamic–pituitary alterations (partial or total, transient or permanent) according to their intracranial localization [12].

Given the sparse nature of the available information on the topic, we propose this scoping review to assess the incidence of endocrinological alterations in patients with prior CNS infection in pediatric age.

## 2. Materials and Methods

We conducted a scoping review including studies aimed at analyzing endocrine complications in pediatric patients with previous CNS infection. 

### 2.1. Review Questions

The main question of our scoping review is “What is known about short-term and long-term endocrine complications in children with previous central nervous system infection?”

Additionally, we analyzed the following secondary questions:

- Is there a difference in the rate of endocrine sequelae between patients with bacterial meningitis caused by different etiological agents?
- Is there a difference in the rate of endocrine sequelae between patients with viral, bacterial, and mycobacterial infections?
- Within bacterial infections, do meningitis and empyemas present a higher rate of endocrine complications compared to localized infections such as abscesses?
- In cases of central origin endocrine complications, is there a correlation with abnormalities in the hypothalamic–pituitary region on radiological examinations performed following the infectious episode?

### 2.2. Inclusion Criteria

All the studies were conducted on children and adolescents (aged 0 to 18 years) with confirmed previous central nervous system infection (clinically diagnosed and/or confirmed by microbiological examinations performed on cerebrospinal fluid). The children included in these studies had experienced a previous episode of meningitis, encephalitis, brain abscess, or intracranial empyema and they underwent subsequent follow-up to evaluate the incidence of hypopituitarism following the acute episode or other endocrinological dysfunctions.

### 2.3. Search Strategy

**Figure 1:**
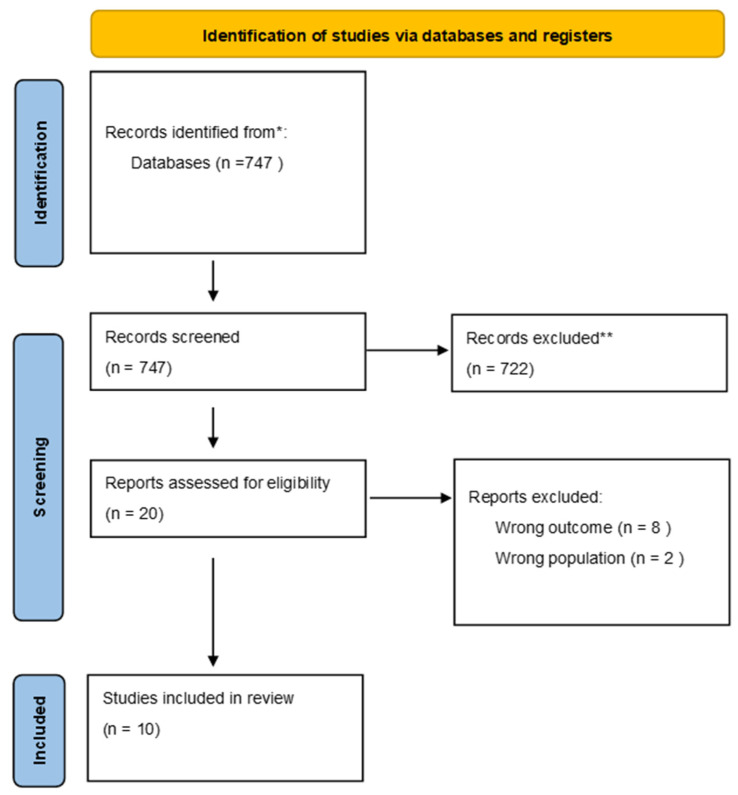
PRISMA 2020 flow diagram for new systematic reviews which included searches of databases and registers only; * PubMed search: ((((((((Central nervous system infections) OR (Meningitis)) OR (encephalitis)) OR (cerebral abscess)) OR (intracranial empyema)) OR (brain empyema)) OR (brain abscess)) AND (((((hypopituitarism) OR (Endocrine)) OR (pituitary function)) OR (endocrine alterations)) OR (hypothyroidism))) AND (((Children) OR (pediatric)) OR (adolescents)). ** Records excluded from our first screening check.

We started our research in February 2024, without data restrictions, in the bibliographic database PubMed, without date restrictions. We included only articles written in English. Our search strategy included the following keywords: central nervous system infections, meningitis, encephalitis, cerebral abscess, intracranial empyema, brain empyema, and brain abscess, and is described in Figure 1. 

Our screening process included both randomized and non-randomized controlled trials. All types of observational studies, prospective and retrospective (including case–control, cohort and cross-sectional studies, and small case series or single case reports) have been included.

### 2.4. Study Selection

Combining the keywords used for our search on PubMed yielded 747 articles (the search strategy appears in the Supplementary Materials). The abstracts of all papers were exported to Rayyan. The article type, title, keywords, and abstract were analyzed by the head researcher to identify articles that might best fit our review. This initial step allowed us to identify and preselect 20 articles, which were subsequently assessed for eligibility by two other reviewers. The full texts of all potentially eligible studies were retrieved. Each researcher was blinded to the decision of the other researcher. Any disagreement between them over the eligibility of studies was resolved through discussion and, in cases of further disagreement, through discussion with a third reviewer. All studies that did not meet the inclusion criteria were excluded. The results of the search were reported in a PRISMA flow diagram.

### 2.5. Data Extraction

Two authors independently extracted the data; an Excel file was used to store the data. When available, extracted information included the following:

- Study general information: title, author, year of publication, type of study, number of patients included in the study, geographical area where the study was performed;
- Participant general features: sample size of each group, nationality, age, socioeconomic status, comorbidities;
- Clinical manifestations of acute CNS infections of children included in our review;
- Type of CNS infection (meningitis, encephalitis, intracranial abscess, and/or empyema);
- Microbiological results (Type of pathogen on liquor analysis);
- Follow-up time and time of onset of endocrinological alterations;
- Type and number of endocrine comorbidities (alterations in GH, TSH, ACTH, LH, FSH, prolactin and desmopressin secretion);
- Main imaging findings (association with brain MRI and/or CT abnormalities);
- Association with other post-infectious deficits (neuromotor deficits, need for tracheostomy and/or PEG, other alterations, death).

### 2.6. Data Analysis and Presentation

We followed the Preferred Reporting Items for Systematic reviews and Meta-Analyses extension for Scoping Reviews (PRISMA-ScR) Checklist [13] (Supplementary Material).

We wrote a narrative synthesis of the findings from the studies included in our review describing the results we obtained and providing our opinion on their interpretation. 

For the narrative synthesis, we preferred articles in which etiological diagnosis was specified; indeed, due to early administration of antibiotics, in some cases, cerebrospinal fluid cultures yielded negative results despite the clinical status of the patients. Furthermore, it is common for brain biopsy to not be performed in encephalitis cases, therefore the pathogen is not always detected in the cerebrospinal fluid.

## 3. Results

Ten studies were included in our review and the general characteristics of the included studies are reported in Table 1. Six studies (60%) were case reports [14,15,16,17,18,19], four of them (40%) were retrospective observational studies [9,20,21,22]. The cumulative number of patients in all of the studies was 211, the mean age of the population study was 4.9 (±5 years). The included papers described the following acute central nervous system infections: meningitis (nine studies reported eighty-five cases) and encephalitis (three studies described sixty-five cases). None of the included studies reported cases of intracranial abscess or empyema. Concerning the pathogens responsible for the infectious diseases, two case reports [14,16] and one retrospective study [21] reported hypopituitarism as a consequence of *Mycobacterium tuberculosis* CNS infection. The bacterial meningitides were caused by *Streptococcus pneumoniae*, *Neisseria meningitidis*, *Streptococcus agalactiae*, *Staphylococcus aureus*, *Haemophilus influenzae* and *Escherichia Coli*. Two retrospective studies [20,22] evaluated the function of the hypothalamic–pituitary axis in children following cerebral bacterial purulent and meningococcal meningitis, without clarifying which pathogens were responsible for the diseases. The patients diagnosed with encephalitis were infected by *Human Parechovirus type 3*, *Enterovirus*, *Varicella Zoster virus*, while in one paper [20] the viral agents were not described. 

In five studies the patients developed endocrine comorbidities at the time of infection. In particular, one neonate [15] was diagnosed with transient hypothyroidism as a consequence of *Human Parechovirus type 3* encephalitis, another one developed Inappropriate Antidiuretic Hormone Secretion following meningitis [19], two case reports described two cases of hypopituitarism due to *M. tuberculosis* and *Streptococcus agalactiae* infections [16,18], respectively, and one paper reported the onset of central diabetes insipidus due to *Escherichia coli* meningitis [17]. In two studies the time of follow-up was not reported. 

Giavoli et al. [9] evaluated the pituitary function after infectious meningoencephalitis in children at a mean follow-up of 18 ± 10 months; they reported the growth parameters and the laboratory results and found no endocrine comorbidities related to CNS infections. Lam et al. [21] assessed the hypothalamic–pituitary functions in 49 young adults who had previously had tuberculous meningitis at a mean age of 5.9 ± 5.0 years: as the authors reported, seven patients had growth hormone deficiency, four of whom also had gonadotropin deficiency; the other three had gonadotropin deficiency, corticotropin deficiency, and mild hyperprolactinemia, respectively; none had diabetes insipidus. 

Interestingly, in our review, hyperprolactinemia as a sequela of infections has been highlighted only by Lam et al. [21], although an increase in prolactin levels is a common occurrence in all inflammatory or expansive processes involving the hypothalamic–pituitary region. Therefore, it is possible that the incidence of hyperprolactinemia is underestimated, as prolactin levels are not generally measured in the follow-up of these patients in the absence of suspicious clinical manifestations. Alternatively, it is possible that prolactin-secreting cells are intrinsically more resistant to inflammatory stimuli and thus are rarely damaged following CNS infections. The details about the mean follow-up time, the type of endocrine comorbidities, the presence of brain MRI abnormalities, and the need of invasive support are reported in Table 2.

The dynamic analyses performed to diagnose hypothalamic–pituitary dysfunction were different. The insulin tolerance test was performed to assess the integrity of the growth hormone (GH) and cortisol axes only in Lam et al.’s paper [21]. In this study, for patients with subnormal cortisol response, further assessment was undertaken using a synacthen (ACTH) test. Giavoli et al. [9] detected baseline ACTH levels and explored the corticotrope axis with synacthen. Otherwise, in Shraga et al.’s study [22] the authors performed a glucagon stimulation test to detect growth hormone and corticotrope deficiency. Summers et al. [14] diagnosed corticotrope insufficiency after the injection of ACTH. 

## 4. Discussion

We conducted this scoping review to analyze the incidence of endocrine dysfunctions in pediatric patients with prior CNS infections. From the results extrapolated from our literature analysis, which is limited by a very low number and quality of studies available on the topic, we highlighted that although hypopituitarism is a rare complication of intracranial infections, endocrine screening should always be performed at a distance from the acute episode to monitor children’s auxological parameters and to identify any signs and symptoms compatible with endocrine system alterations.

Hypopituitarism, especially when partial, can remain asymptomatic for a long time and manifest later [23]; this is the case, for example, with short stature due to growth hormone deficiency [24], in which damage to somatotropic cells may not cause acute alterations but can manifest with reduced growth velocity and loss of height percentiles [23]; other possible manifestations are seizures or hypoglycemia [25] and, during adulthood, reduced female fertility due to decreased maturation of granulosa cells and reduced modulation of FSH and LH [26].

Gonadotropin deficiency becomes evident mainly during the peripubertal period and can manifest with lack of progression of secondary sexual characteristics, but they can also be diagnosed during the mini-puberty period due to unmeasurable levels of LH [27]. TSH deficiency causes central hypothyroidism [28], usually symptomatic with the onset of lethargy, fatigue, weight gain, and metabolic manifestations such as hypercholesterolemia, while prolactin deficiency and/or hypersecretion, despite their relationship with growth hormone secretion, is often asymptomatic in pediatric age [29]. The most severe cases manifest with ACTH deficiency and central adrenal insufficiency, a serious condition that puts children’s lives at risk [30]. Involvement of the neurohypophysis causes diabetes insipidus with polyuria, polydipsia, and alterations in sodium levels and plasma osmolarity, and is generally symptomatic [31].

**Figure 2:**
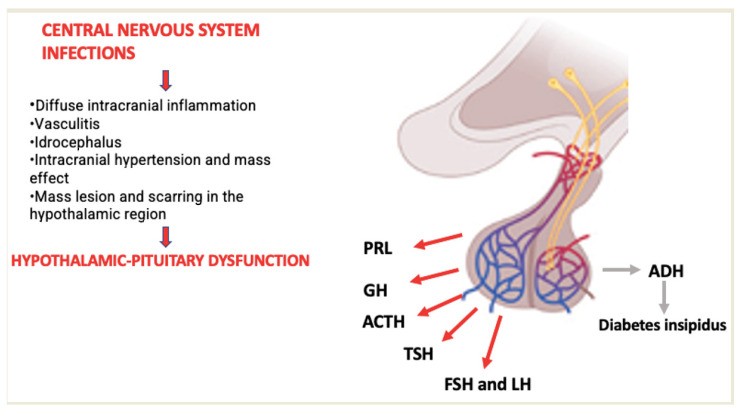
Pathogenetic mechanisms of endocrine dysfunctions.

A timely diagnosis of all these alterations is necessary because for each endocrine defect there is a replacement or supplementary hormone therapy capable of restoring the normal functionality of the organism, regardless of the cause of the hypothalamic–pituitary lesion. This is exemplified by the hormone replacement therapies for oncological patients, in whom CNS damage is caused by the tumor or the medical treatments used [32]. Similarly, in infectious processes, intracranial inflammation can lead to alterations of the hypothalamic–pituitary axis and the deficiency of specific pituitary tropic hormones [10]. We illustrate in Figure 2 the main pathogenetic mechanisms by which central nervous system infections cause endocrine dysfunctions.

Regarding the secondary questions we posed, within the context of bacterial meningitis, we highlighted how it is not known which bacterium is most frequently associated with hypothalamic–pituitary alterations due to the heterogeneity of the studies analyzed; in real-world experience, data about mortality and morbidity have shown that Streptococcus pneumoniae is responsible for 17.3% of deaths caused by CNS infection in children under 5 years old, despite being third in incidence of intracranial infections (13.3%) and second in bacterial infections (after meningococcus) [2]. Analyzing the subgroup of neonates, the pathogen associated with the highest mortality rate is group B Streptococcus (22.8%) [2], which is explained by the fact that it is a bacterium that frequently colonizes the vaginal canal and, if not adequately treated, can cause invasive infections in newborns [33]; indeed, this trend persists even in older children, potentially causing complications such as ischemic or hemorrhagic brain lesions [34]. 

A systematic review of pediatric guidelines in recent years highlights that initial empirical therapy should take into account the epidemiological characteristics of the geographic region. European guidelines suggest monotherapy with benzylpenicillin, ceftriaxone, or cefotaxime and the addition of corticosteroids in cases of suspected pneumococcal or Haemophilus influenzae etiology [35]. Despite the growing evidence that many pediatric infections can be treated with personalized-duration antibiotic regimens [36], the antibiotic therapy for meningoencephalitis is an exception, as it requires prolonged and intravenous administrations to improve outcomes and reduce the risk of short- and long-term complications.

Despite adequate antibiotic therapy, given the invasive nature of these infections and their high mortality rate, residual brain alterations often persist, which can lead to seizures, hydrocephalus, and other neurological abnormalities; the neuropsychiatric follow-up of these patients is generally standardized [37], while the endocrinological follow-up is not standardized in most cases and is not always performed unless there is a high suspicion of hypothalamic–pituitary alterations.

Schaefer et al. [38] analyzed 19 patients (38.7 ± 11.7 years) with a previous diagnosis of central nervous system infection, evaluating the basal pituitary reserve stimulated by the insulin tolerance test. The analysis of basal tests showed normal functionality of the somatotropic, thyrotropic, and neurohypophyseal axes in all cases. However, four cases of corticosurrenal deficiency and two cases of borderline hypogonadotropic hypogonadism (testosterone levels below the normal range) were found. In all cases, cortisol deficiency was subclinical and associated with chronic fatigue without acute symptoms of corticosurrenal insufficiency, which improved following hydrocortisone replacement therapy in only one patient. One of the patients with hypogonadism presented erectile dysfunction, while the other was asymptomatic. The authors concluded that many of the symptoms attributed to sequelae of the infection could be related to corticotropin deficiency, partial or complete, which should therefore be evaluated in the follow-up of these patients. However, the authors did not perform a subgroup analysis to assess the incidence of dysfunctions based on the etiological agent causing the infection. 

Tsiakalos et al. [39] conducted a similar study, analyzing pituitary function in 16 adult patients within 24 h of hospitalization and one year after discharge. In five cases (two viral infections and three bacterial meningitis), subclinical endocrinological alterations were found at one year post-discharge (two corticotropin deficiencies, one somatotropin deficiency, and two multiple combined deficiencies). Four out of five patients had normal pituitary function at admission, while only one (with somatotropin deficiency) had persistent deficiency. The results of this study contribute to reinforcing the hypothesis that hypopituitarism, even if paucisymptomatic, should be sought in patients with risk factors such as central nervous system infections. The acute alterations (five patients, 31.25%) evaluated by the authors are less significant since it is known that transient hormonal alterations, such as those related to thyroid function, can occur during acute inflammatory states [40].

Our literature analysis has shown that hypopituitarism and endocrine complications are not typical of viral infections, whereas they are more commonly associated with bacterial and mycobacterial infections. Indeed, in the literature, there are no reported cases of pediatric patients with endocrinological dysfunctions following infectious encephalitis [41]. However, when extending the research to the adult population, rare cases of diabetes insipidus or complete hypopituitarism are reported as sequelae of severe herpes virus encephalitis; the endocrinological consequences of viral encephalitis remain exceptional events, likely due to less frequent anatomical sequelae compared to bacterial infections [37]. It is important to highlight that the available literature on the endocrinological consequences of viral infections is scarce and this can explain why the endocrine deficits may seem rare.

*Mycobacterium tuberculosis*, when causing meningoencephalitis, often leads to pituitary involvement with the onset of multiple tropic deficits and hyperprolactinemia due to intracranial inflammatory complications secondary to the infection [42]. Lam et al. [21] analyzed forty-nine young adults (aged 23.4 ± 6.0 years) with a history of tuberculous meningitis during childhood, highlighting how 20% (10/49) developed at least one tropic deficit upon evaluation (seven growth hormone deficiencies, four of whom also had gonadotropin deficiency; the other three cases had gonadotropin deficiency, corticotropin deficiency, and mild hyperprolactinemia). An MRI analysis of the pituitary region revealed anomalies in only five out of ten patients (50%), demonstrating that the pathogenesis of post-infectious hypopituitarism is multifactorial and that a normal radiological examination cannot rule out an underlying hormonal deficit. The true incidence of pituitary dysfunction following tuberculous meningitis is unknown because the associated pituitary manifestations are variable and unpredictable, often remaining latent for years after the primary infection [43].

Comparing patients with bacterial meningitis and those with brain abscesses, we have highlighted that the localization and involvement of the hypothalamic–pituitary region remain fundamental factors in the development of hypopituitarism. Generally, meningitis can cause pituitary alterations due to diffuse intracranial inflammation, while intracranial abscesses can lead to hypopituitarism when localized to the pituitary region and can cause symptoms of intracranial hypertension and mass effect before endocrinological disturbances occur [44].

Finally, evaluating the results of radiological examinations of patients included in our review, we highlighted that the onset of hypopituitarism is closely correlated with the presence of post-inflammatory anatomical anomalies found on brain magnetic resonance imaging, particularly those affecting the pituitary region. A recent study [45] has also highlighted that post-infectious pituitary dysfunction may also have an autoimmune origin due to the emergence of anti-pituitary and anti-hypothalamus antibodies; however, the relationship is not clear, and in the analyzed sample there is no statistically significant correlation with clinical hypopituitarism.

All the studies analyzed in our review examined the correlation with involvement of the hypothalamic–pituitary organ, and in six cases have shown brain MRI abnormalities following the acute phase of infection. It is unclear whether the acute diencephalic involvement is associated with possible subsequent endocrinological complications, and further studies could explore this association.

In conclusion, our scoping review highlights that there is a paucity of research regarding the long-term endocrinological outcomes of children with CNS infections, particularly following viral infections. In addition, most cohorts are variegated in terms of disease or age onset of the infection, therefore limiting the possibility to derive conclusions based on specific pathogens and age groups. Despite these limitations, it appears clear that bacterial and mycobacterial infections in particular can lead to subtle long-term endocrine deficits that may remain long overlooked if not actively searched for. Therefore, standardized multidisciplinary follow-up and research of patients with prior central nervous system infection is crucial. Although pituitary reserve screening is not commonly performed on these patients, clinical and research centers should set up an endocrinological evaluation with monitoring of auxological parameters to detect signs and symptoms of hypopituitarism early and to initiate appropriate care in children with previous CNS infections.

## Figures and Tables

**Table 1:** Characteristics of the included studies.

Author	Year of Publication	Type of Study	Aim of the Study	Number of Patients	Age	Type of Infection	Type of Path.
V.K. Summers [14]	1968	Case report	To describe a case of Panhypopituitarism after cured tuberculous meningitis	1	6 years old	Meningitis	* M. tuberculosis *
A. Dereymaeker [15]	2015	Case report	To describe a case of transient hypothyroidism associated with viral Human Parechovirus encephalitis in a newborn	1	9 days old	Encephalitis	Human Parechovirus type 3 (HPeV-3)
C. Giavoli [9]	2014	Retrospective study	To determine the incidence of pituitary dysfunction in children with central nervous system infections	19	5.9 ± 4.0 years old	11 patients had meningitis 8 patients had encephalitis	*Enterovirus*, Varicella Zoster virus, *Streptococcus pneumoniae*, *Neisseria meningitidis*, *Streptococcus agalactiae*, *Staphylococcus aureus*, *Haemophilus influenzae*, *Mycobacterium tuberculosis*
B. Indira [16]	1996	Case report	To describe a case of hypopituitarism secondary to tuberculoma of the hypothalamic region	1	10 years old	Meningitis	*M. tuberculosis*
Fuyong Jiao [20]	2011	Retrospective study	To find the changes of thyroid hormone in serum and/or CSF in relation to CNS infections	123	Not known	67 patients had meningitis, 56 patients had encephalitis	46 patients had bacterial meningitis (purulent meningitis, meningococcal meningitis) 56 patients had mycobacterial meningitis (*M tuberculosis*) 21 patients had viral meningitis
K. S. L. Lam [21]	1993	Retrospective study	To study the prevalence and pathogenesis of hypopituitarism following tuberculous meningitis in childhood	49	23.4 ± 6 years old	Meningitis	*M. tuberculosis*
Yael Levy-Shraga [22]	2013	Retrospective study	To evaluate pituitary function of children with a history of meningitis.	14	3.8 ± 5.4 years	Meningitis	3 patients had bacterial meningitis
S. Mizuno [17]	2023	Case Report	To describe a case of Waterhouse–Friderichsen Syndrome and central diabetes insipidus due to *E. coli* sepsis in a newborn	1	1 day	Meningitis	* E. coli *
A. S. Ferreira [18]	2015	Case Report	To describe a case of a 39-day-old infant with meningitis caused by Streptococcus Group B, which showed, among other consequences, hypopituitarism.	1	1 day	Meningitis	Streptococcus Group B
D. W. Reynolds [19]	1972	Case Report	To describe a case of Inappropriate Antidiuretic Hormone Secretion in a newborn with meningitis.	1	9 days old	Meningitis	*E. coli*

**Table 2:** Endocrine comorbidities following acute CNS infection.

Author	Time of Onset of Endocrine Comorbidities	Type of End. Com.	Symptomatic Deficit	Association of Brain MRI Abnormalities	Need of Invasive Support (Ventilation, Trachea, PEG)	Summary of Results
VK Summers [14]	14 months	Short stature (145 cm at the age of 20); amenorrhea; hypothyroidism; hypogonadotropism; hypoadrenalism	Yes	MRI not performed; suprasellar calcifications at X-ray	no	Short stature; amenorrhea; hypothyroidism; hypogonadotropism; hypoadrenalism
A. Dereymaeker [15]	At the time of infection	Transient hypothyroidism	Yes	Yes (areas of restricted diffusion in the periventricular white matter, corpus callosum, deep white matter, optic radiation, internal capsule and both thalami)	no	Transient hypothyroidism
C. Giavoli [9]	18 ± 10 months	No comorbidities	No	Not known	Not known	All of the subjects had a normal stature; none of the subjects had central hypothyroidism; all of the patients had normal serum of IGF-I (mean IGF-I SDS ± SD −0.9 ± 0.5) and prolactin, and their sex steroid and gonadotropin levels were concordant with their age and pubertal status; no signs of diabetes insipidus; all of the patients had normal plasma ACTH levels
B. Indira [16]	At the time of infection	Hyperphagia, weight gain, excessive somnolence, and apathy secondary to hypothyroidism and hypoadrenalism	Yes	Yes (compression of the third ventricle with hydrocephalus)	no	hyperphagia, weight gain, excessive somnolence and apathy secondary to hypothyroidism and hypoadrenalism
Fuyong Jiao [20]	Not known	Not known	Not known	Not known	Not known	(1) the mean T3 and T4 in serum and the mean T4 in CSF were significantly lower in children with severe CNS infections than that in healthy adults (*p* < 0.05); (2) The decrease of CSF T4 was higher than that of T3 in children with severe CNS infections; (3) the children with low T3 showed lower survival rate than those with low T4. The data have shown that T3 and T4 in serum are more decreased in severe infections of CNS than in mild
K. S. L. Lam [21]	17.5 ± 5. 2 y. after the infection	Growth hormone deficiency, gonadotropin deficiency, mild hyperprolactinemia	yes	Yes (enhanceable lesions in the hypothalamus, pituitary stalk, or suprasellar cistern; dilatation of the third ventricle due to aqueduct stenosis; adhesion in the basal arachnoid or focal thalamic atrophy; pituitary atrophy of varying severity)	no	Ten patients were found to have abnormal pituitary function: Seven had growth hormone deficiency, (The height SDS of the patients at the time of study correlated positively with the age at meningitis (r = 0.749, *p* < 0.05).) four of whom also had gonadotropin deficiency; the other three had gonadotropin deficiency, corticotropin deficiency, and mild hyperprolactinemia, respectively; none had diabetes insipidus.
Yael Levy-Shraga [22]	/	None of them developed pituitary hormone deficiencies	/	/	/	None of them developed pituitary hormone deficiencies
S. Mizuno [17]	At the time of infection	Central diabetes insipidus	yes	Yes (bilateral encephalomalacia, minor bleeding, and a splenial lesion)	Not known	Central diabetes insipidus
A. S. Ferreira [18]	At the time of infection	Central diabetes insipidus, adrenocortical insufficiency, and central hypothyroidism	yes	Yes (extensive diffuse inflammatory/infectious involvement of the meninges with impaired signals associated with underlying brain parenchyma, especially in frontal region, left nucleocapsular region, midbrain, and pons. Subdural collections of thick content, not hemorrhagic, in right frontoparietal region and left frontal region)	Yes	Hypopituitarism secondary to CNS infection by GBS
D. W. Reynolds [19]	At the time of infection	SIADH	yes	Not known	Not known	Central nervous system disorders presumably initiate inappropriate ADH release by direct irritation of the supraoptic and paraventricular nuclei or hypophyseal tract or both, or by interference with normal neuronal in flow to these areas.

## Supplementary Materials

The following supporting information can be downloaded at: https://www.mdpi.com/article/10.3390/children11070794/s1, Table S1: Preferred Reporting Items for Systematic reviews and Meta-Analyses extension for Scoping Reviews (PRISMA-ScR) Checklist.
